# Detection of Sub-Micro- and Nanoplastic Particles on Gold Nanoparticle-Based Substrates through Surface-Enhanced Raman Scattering (SERS) Spectroscopy

**DOI:** 10.3390/nano11051149

**Published:** 2021-04-28

**Authors:** Jessica Caldwell, Patricia Taladriz-Blanco, Barbara Rothen-Rutishauser, Alke Petri-Fink

**Affiliations:** 1 Adolphe Merkle Institute, University of Fribourg, Chemin des Verdiers 4, 1700 Fribourg, Switzerland; jessica.caldwell@unifr.ch (J.C.); barbara.rothen@unifr.ch (B.R.-R.); 2 Department of Chemistry, University of Fribourg, Chemin du Musée 9, 1700 Fribourg, Switzerland

**Keywords:** nanoplastic, sub-microplastic, Raman, SERS, nanoparticles

## Abstract

Small plastic particles such as micro- (<5 mm), sub-micro- (1 µm–100 nm) and nanoplastics (<100 nm) are known to be ubiquitous within our surrounding environment. However, to date relatively few methods exist for the reliable detection of nanoplastic particles in relevant sample matrices such as foods or environmental samples. This lack of relevant data is likely a result of key limitations (e.g., resolution and/or scattering efficiency) for common analytical techniques such as Fourier transform infrared or Raman spectroscopy. This study aims to address this knowledge gap in the field through the creation of surface-enhanced Raman scattering spectroscopy substrates utilizing spherical gold nanoparticles with 14 nm and 46 nm diameters to improve the scattering signal obtained during Raman spectroscopy measurements. The substrates are then used to analyze polystyrene particles with sizes of 161 nm or 33 nm and poly(ethylene terephthalate) particles with an average size of 62 nm. Through this technique, plastic particles could be detected at concentrations as low as 10 µg/mL, and analytical enhancement factors of up to 446 were achieved.

## 1. Introduction

As a result of the degradation of products composed of synthetic polymers such as polystyrene (PS), poly(ethylene terephthalate) (PET), polypropylene (PP), and polyethylene (PE) (e.g., food and beverage packaging), small particles, commonly referred to as micro- (<5 mm), sub-micro- (1 µm–100 nm) and nanoplastics (<100 nm), are highly prevalent within our surrounding environment. Studies have reported the presence of microplastic particles within various products meant for human consumption [1,2] (e.g., due to the previous ingestion of plastics by fish and shellfish meant for consumption) as well as in samples collected from oceans [3], soils [4], and the atmosphere [5] worldwide. However, as a result of their exceptionally small size and low estimated concentrations within such environments [6], studies which report the presence of sub-micro- and nanoplastic particles are limited [7].

Among the techniques most commonly utilized within studies which quantify plastic particle presence in food and environmental samples, Raman spectroscopy is one of the most promising for the detection of sub-micro- and nanoplastic particles due to its reported viability for smaller plastic particles (i.e., microplastics ~1 µm or larger in size [8]) compared to other techniques such as stereomicroscopy (i.e., microplastics ~500 µm or larger in size [9]) or Fourier transform infrared spectroscopy (FTIR) (i.e., microplastics ~10 µm or larger in size [8]). However, due to the diffraction limited instrument resolution for optical microscopy setups (i.e., half the wavelength of the excitation light utilized) and the low probability of spontaneous Raman scattering (i.e., 1 in 10^8^ of the incident radiation undergoes Raman scattering), conventional Raman spectroscopy is not sensitive enough to address the issue of sub-micro- and nanoplastic particle detection without further modifications [10,11,12].

One such modification used to overcome the current analytical limitations of Raman spectroscopy is the technique knowns as surface-enhanced Raman scattering (SERS) spectroscopy, which involves placing the sample of interest in close proximity to or adsorbing it to a plasmonic metal surface; predominantly gold (Au) and silver (Ag) [13,14,15]. This technique takes advantage of the plasmonic oscillations of the surface electrons surrounding the metal surface to enhance the inelastic Raman scattering signal of the analyte, with enhancement factors of up to 10^14^–10^15^ reported for optimized systems [13].

Despite such promising signal improvements being reported for other systems, to date relatively little literature exists which discusses the use of the SERS for the detection of sub-micro- or nanoplastic particles. Xu et al. have reported the use of a commercially available Klarite substrate to detect particles composed of PET, PS, and poly(methyl methacrylate) (PMMA) with signal enhancements of up to 2 orders of magnitude [16]. However, the particles analyzed in this study were all reported to be >360 nm in size [16]; thus exceeding the maximum size (i.e., 100 nm) defined for a nanomaterial by the European Commission [17]. In a study conducted by Lv et al. it was reported that the detection of PS particles with a diameter of 100 nm was possible, and that the enhancement factors observed could be as much as 500 times greater than a regular Raman spectroscopy signal [18]. This enhancement was achieved through the aggregation of silver nanoparticles (AgNPs) with salts [18]. Thus, there is still a great need in the field for cheap, reproducible SERS substrates that can be used to detect sub-micro- or nanoplastic particles; particularly when the sample of interest contains a very low concentration of plastics (e.g., more environmentally relevant concentrations on the order of nanograms) [6].

This study aims to address this gap in the field through using SERS substrates fabricated through the layer-by-layer technique. The high potential of SERS as an analytical technique is strictly linked to the development of highly reproducible and reliable substrates. Therefore, when using colloidal nanoparticles as SERS substrates, control over their synthesis is required [19]. However, the preparation of highly reproducible monodisperse AgNPs is much more difficult than for gold nanoparticles (AuNPs) due to the higher reactivity of Ag atoms than Au atoms [20]. In addition, it has been reported in the literature that AgNPs undergo dissolution in aqueous solutions, which limits their applicability as SERS substrates, especially for environmental samples [21]. Based on this, despite the larger scattering contribution and thus higher SERS enhancement factor of AgNPs compared to AuNPs, this study was conducted utilizing 14 nm and 46 nm spherical AuNPs. As a proof of concept, PS particles with sizes of 161 or 33 nm and PET particles with a size of 62 nm were detected with concentrations as low as 10 µg/mL.

## 2. Materials and Methods

### 2.1. Materials

Tetrachloroauric acid (HAuCl_4_∙3H_2_O, ≥99.9%), sodium citrate tribasic dihydrate (C_6_H_5_Na_3_O_7_∙2H_2_O, ≥99.5%), sodium dodecyl sulfate (SDS; ACS reagent; NaC_12_H_25_SO_4_; ≥99.9%), poly(allylamine hydrochloride) (PAH; [CH_2_CH(CH_2_NH_2_·HCl)]_n_; average Mw 17,500), and styrene (ReagentPlus^®^ reagent; C_8_H_8_; ≥99.9%) were purchased from Sigma-Aldrich, Buchs, Switzerland. Hydroxylamine hydrochloride (NH_2_OH∙HCl, ≥99.0%) and potassium peroxodisulfate (KPS; ACS reagent; K_2_S_2_O_8_, ≥99.9%) were purchased from Fluka, Buchs, Switzerland. Hydrogen peroxide (H_2_O_2_, 30 wt.% in H_2_O) was purchased from Reactolab SA, Servion, Switzerland. Sulfuric acid (ISO + Ph. Eur. Reagent; H_2_SO_4_, ≥95%) was obtained from Honeywell, Regen, Germany. PET ([C_10_H_8_O_4_]_n_) pellets were purchased from Goodfellow Cambridge Ltd., Huntingdon, UK. All water was purified with an 18.2 MΩ.cm arium 611DI MilliQ system (Sartorius Stedim Biotech, Göttingen, Germany) prior to use.

### 2.2. Preparation of Gold and Plastics Particles

Sub-micron PS particles with a 161 nm diameter were prepared by adding 16.1 g of styrene to an SDS solution (149 mg SDS dissolved in 59 mL of MilliQ water under 300 rpm stirring) previously degassed with N_2_. The final emulsion was degassed with N_2_ and heated to 60 °C for 1 h. The temperature was then increased to 80 °C for 45 min before 3.33 mL of a KPS stock solution (195 mg in 20 mL of MilliQ water) was introduced dropwise over the course of 10 min. The reaction was stirred at 80 °C overnight. To purify, particles were dialyzed using a 14 kDa cutoff membrane (Carl Roth GmbH + Co, Arlesheim, Switzerland) for 3 weeks.

PS particles with a 33 nm diameter were synthesized by mixing 2 g of styrene with 480 mg SDS and 11 mL of MilliQ water under 300 rpm stirring. The final emulsion was degassed with N_2_ and heated to 75 °C for 30 min prior to the dropwise addition of 1 mL of a KPS solution (16.6 mg in 1 mL of MilliQ). The reaction was then stirred at 75 °C under N_2_ flux for 5 h. To purify, particles were dialyzed using a 14 kDa cutoff membrane (Carl Roth GmbH + Co, Arlesheim, Switzerland) for 1 week.

For both types of PS particles, stock concentrations were acquired by mass balancing the dried particle powders obtained from a fixed (1 mL) volume using an AG204 Delta Range balance (Mettler-Toledo GmbH, Greifensee, Switzerland). This process was repeated 6 times to obtain the final, average stock concentration.

PET particles with an average diameter of 62 nm were prepared using the sequential milling method previously described by Caldwell et al. [22]. Briefly, PET pellets were milled under liquid-nitrogen cooling with a 6770 Freezer Mill (steel milling rod; steel chamber plugs; polycarbonate chamber; SPEX, Metuchen, NJ, USA) prior to milling at 15 °C in a NanoWitt-Lab mill (zirconium dioxide beads; FREWITT SA, Granges-Paccot, Fribourg, Switzerland). To purify, milled particle dispersions were run through a Chromafil filter with a pore size of 0.2 µm (Macherey-Nagel, Düren, Germany) and dialyzed in a membrane with a 14 kDa cutoff. Complete production and characterization details for the milled PET particles have been reported previously [22].

AuNPs with a 14 nm diameter were prepared via the Turkevich method [23]. Briefly, an aqueous solution of HAuCl_4_ (0.5 mM) was brought to reflux and mixed with 1.7 mM sodium citrate for 15 min. The solution was then allowed to cool to room temperature while stirring prior to UV-vis characterization. The dispersion was kept in the fridge until further use.

AuNPs with a 46 nm diameter were prepared via the Brown method with slight modifications [24]. Briefly, 0.0125 mM of as-prepared 14 nm AuNPs were added to an aqueous solution of HAuCl_4_ (0.25 mM) and of sodium citrate (0.5 mM) under magnetic stirring. A solution of NH_2_OH∙HCl (1.96 mM) was then added, and the reaction was left to stir for 15 min. Particles were cleaned by centrifugation for 20 min at 3500 rpm and concentrated in a 1 mM sodium citrate solution to obtain a final concentration of 1 mM Au^0^. The dispersion was kept in the fridge until further use.

### 2.3. Fabrication of SERS Substrates

Glass microscopy slides (Thermo Fisher Scientific, Bremen, Germany) were cleaned by soaking them 30 min in a piranha solution (10 mL of H_2_O_2_ with 30 mL of H_2_SO_4_), rinsing with MilliQ water, and drying with N_2_. Dried slides were then soaked in 40 mL of a 40 mg/mL solution of PAH for 15 min. The excess polyelectrolyte was removed by rinsing the slides with MilliQ water and drying with N_2_ prior to a 4 h soak in 40 mL of AuNP dispersion. Finally, the Au-functionalized glass slides were rinsed with MilliQ water prior to drying in air at room temperature overnight. A 40 mL aliquot of PAH and 40 mL of AuNPs could be utilized to create a batch of ~4 glass slide substrates in one run; thus, multiple substrates were created and utilized for SERS measurements. In addition, substrates were created using three different batches of nanoparticles prepared on different weeks/months.

### 2.4. Characterization of AuNPs, PS and PET Particles, and SERS Substrates

UV-vis extinction spectra of AuNPs in water were recorded at room temperature (RT) with a V-670 spectrophotometer (Jasco, Oklahoma City, OK, USA) using 10 mm path length quartz suprasil cuvettes (Hellma Analytics, Müllheim, Germany). To obtain UV-vis spectra of the substrates, baseline correction was performed with clean glass slides prior to the AuNP-functionalized glass slides being placed in the measurement pathway for spectra collection at RT.

A Tecnai Spirit transmission electron microscope (TEM, FEI, Columbia, MD, USA) operating at 120 kV was used to image the AuNPs. A 10 µL drop was cast onto carbon film on copper 300 square mesh (Electron Microscopy Sciences, Pennsylvania, PA, USA) and dried at RT before visualizing the particles with a 2048 × 2048 pixel wide angle Veleta CCD camera (Olympus, Toyko, Japan). TEM images were processed with the ImageJ software (v1.52). Average AuNP size and standard deviation were measured manually in Fiji (ImageJ, Wayne Rasband National Institute of Health, Bethesda, MD, USA [25]).

Hydrodynamic diameters and zeta potentials of the particles were measured using a 90Plus Particle Size Analyzer (Brookhaven Instruments Corporation, Holtsville, NY, USA) with phase-amplitude light scattering (PALS) for zeta-potential determination (Brookhaven Instruments, Holtsville, NY, USA) at an angle of 90° with a 40 mW diode laser, λ = 640 nm. The analysis was carried out in diluted suspensions in MilliQ water at RT. The hydrodynamic diameter of the PET particles was additionally obtained with a commercial goniometer instrument (3D LS Spectrometer, LS Instruments AG, Fribourg, Switzerland) by averaging multiple measurements taken in 10° steps from 30° to 150° with 10 measurements of 30 s taken per angle. The stock concentration was measured through mass balancing with an OpenQCM quartz crystal microbalance (QCM; Novaetech S.r.l., Frascati, Italy).

PS and PET particles were imaged using a scanning electron microscope (SEM, TESCAN Mira 3 LM field emission, Kohoutovice, Czech Republic). Briefly, 10 µL of diluted stock particles were dried on glass slides affixed to aluminum SEM stubs (Agar Scientific, Stansted, UK) with carbon black tape (Agar Scientific, Stansted, UK) and sputter coated with a 1 nm thick layer of gold using a 208 HR sputter coater (Cressington Scientific Instruments, Watford, UK). Average particle size and standard deviation were measured manually in Fiji. AuNP substrates were also imaged with an SEM. Control images of clean substrates were obtained by mounting the slides to aluminum SEM stubs with carbon black tape. The edges of the mounted slides were coated with conductive silver paste (Plano GmbH, Wetzlar, Germany) prior to sputter coating with a 1 nm thick layer of gold. Additional images were obtained in the same manner for the substrates once plastic particles were drop-cast onto their surface.

### 2.5. Raman and SERS Spectroscopy

All Raman and SERS measurements were conducted with a WiTec Alpha 300 R confocal Raman microscope operated with a 785 nm laser wavelength, a 50× magnification objective, and a built-in CCD camera for obtaining bright field images (WITec, Ulm, Germany). Individual SERS spectra were collected by accumulating multiple (i.e., 25–300) 1 s scans to generate a final, average spectra. Laser power ranged from 2 mW to 45 mW depending on the sample and the substrate used. Exact measurement details are given in Table A1. Automated SERS maps were collected by acquiring single 1 s spectra at multiple points within a defined region of interest (ROI). Laser power for mapping ranged from 2 mW to 7 mW depending on the sample. Confocal Raman image processing was conducted with the accompanying WITec Control 5 software, and all chemical fingerprint data was baseline corrected with this software (i.e., the measured background spectrum of each slide was subtracted from the sample data collected (Appendix A), a shape subtraction filter of 300 was applied, cosmic ray filters were applied).

### 2.6. Contamination Prevention

In addition to washing glass slides with piranha to prevent contamination from previously deposited organic matter, slides were stored in closed containers during all procedural steps which did not directly involve their handling, and during the time between sample creation and analysis. (Disclaimer: piranha solution is a strong oxidizing substance and must be prepared with care.) When samples were handled, cotton lab coats and latex gloves were worn. All glassware was pre-washed prior to contact with a sample of interest.

## 3. Results

### 3.1. SERS Substrates

**Figure 1:**
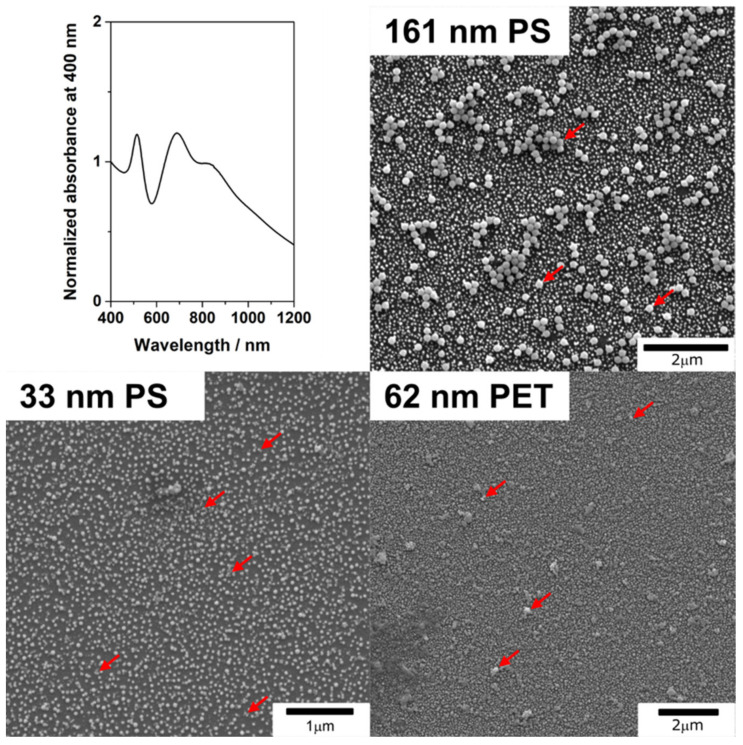
UV-vis extinction spectra of the 46 nm AuNP substrates and representative SEM images of the 161 nm PS, 33 nm PS, and 62 nm PET particles after their addition on top of the substrates. These images were taken at the middle of the cast drop and show that the gold and plastic particles are distributed homogenously throughout the substrate surface. Several examples of plastic particles are indicated with red arrows for clarity. See Figure A3 for additional SEM pictures.

**Figure A1:**
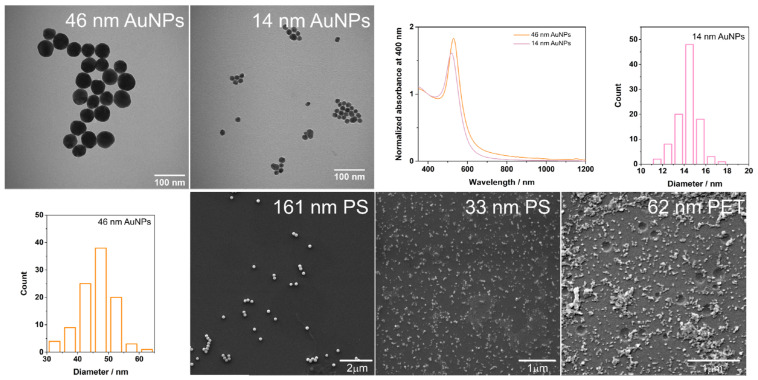
UV-vis extinction spectra for the stock AuNP dispersions are shown alongside representative TEM images for the 46 nm gold and the 14 nm gold stock particles as well as histograms of their sizing data. SEM images for the 161 nm PS, the 33 nm PS, and the PET are also shown.

**Figure A2:**
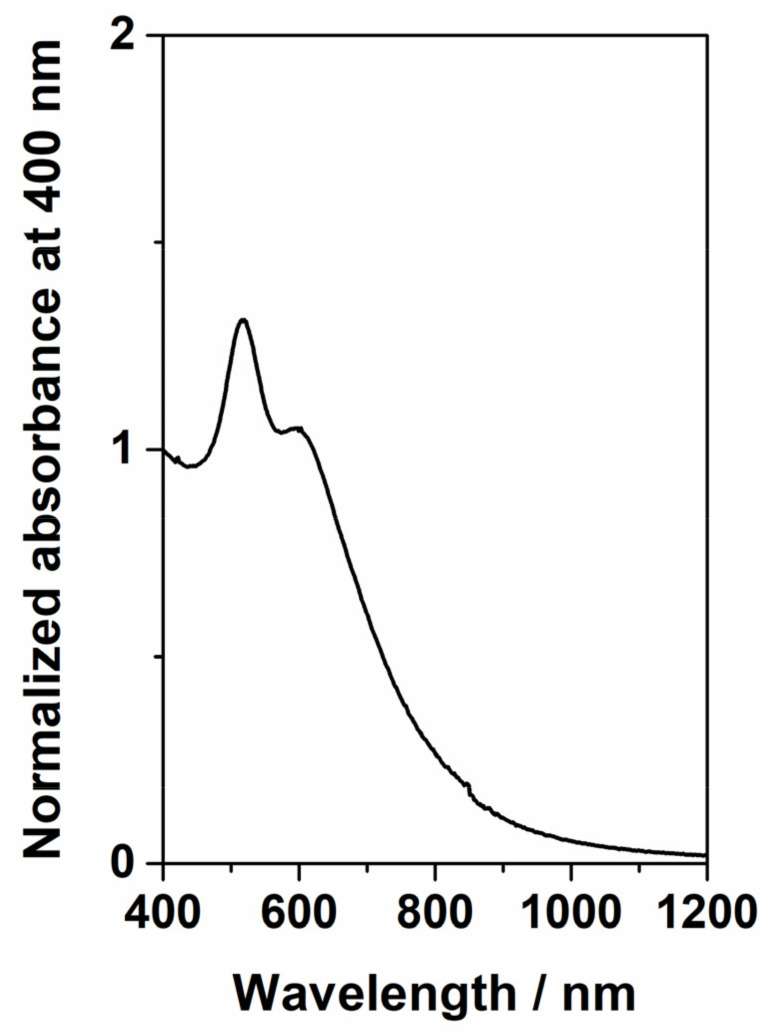
Representative extinction spectra for the 14 nm AuNP substrates.

SEM images of the SERS substrates were successfully created via electrostatic layer-by-layer assembly utilizing a positively charged polyelectrolyte and negatively charged AuNPs in a manner that has been previously reported and validated in the literature [26,27,28]. Through soaking the glass slides in PAH and then in either citrate-stabilized 46 nm or 14 nm AuNPs, (see Figure A1 and Table 1 for the physicochemical characterization) a homogeneous distribution of the AuNPs on the final substrate could be obtained (Figure 1) [28]. The 46 nm AuNP substrate featured a plasmon band at 520 nm corresponding to the dipole resonance of individual AuNPs and two broad bands at 690, and 836 nm which correspond to the plasmon coupling between NPs in close proximity (Figure 1). Similarly, the 14 nm AuNP substrates exhibit two bands centered at 519 and 598 nm, respectively (Figure A2).

**Figure A3:**
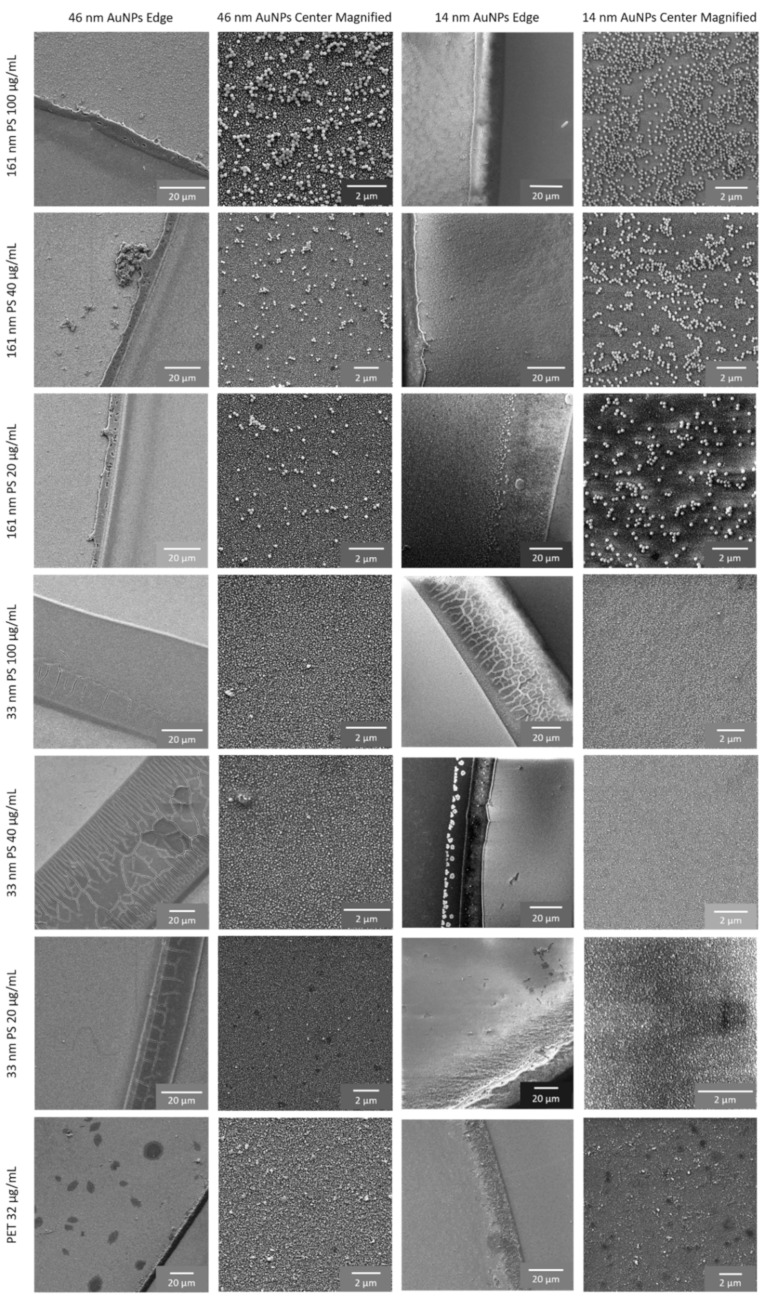
SEM images for the final, dried plastic particle samples on the SERS substrates. Image columns are labelled with the gold nanoparticle type used to create the substrate. Image rows are labelled with the type of plastic particle sample represented. 20 μm scale images show the dried edge of the plastic samples. 2 μm images are obtained from the sample centers.

PS particles in the sub-micron (161 ± 19 nm) and the nano (33 ± 9 nm) size range, in the following referred as to 161 nm PS and 33 nm PS particles, and PET particles with a core size of 62 ± 38 (Table 1), were cast onto the substrates and dried (Figure 1 and Figure A3). In SEM images the plastic particles are observed to be distributed on the substrates following the “coffee-ring” effect (Figure A3); with a more homogenous layer of plastic particles present at the center of the dried sample drop surrounded by a ring of particle buildup. This was observed for all particles, down to the lowest concentrations studied and is an effect that has been studied extensively in the literature [29,30,31].

### 3.2. SERS Detection of 161 nm PS, 33 nm PS, and 62 nm PET Particles

**Figure A4:**
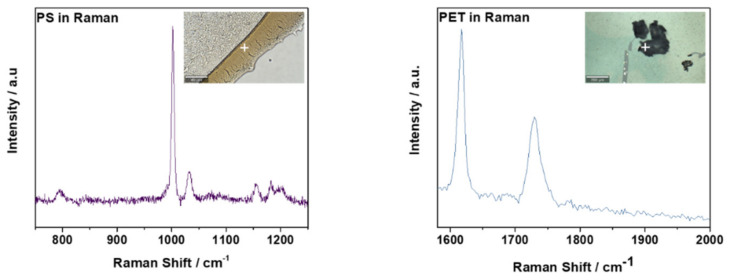
Representative Raman spectra measured for 161 nm PS sub-microparticles which were drop-cast in layers until a thick enough deposition was achieved to obtain a Raman signal (100× magnification; scale bar 40 µm) (left) and for stock milled PET microparticles (average size <170 µm [22]; scale bar 200 µm). Laser power, scan speeds, and number of averaged accumulations for the samples can be viewed in Table A1. Spectrum obtained are shown with the region of interest (ROI) the measurement was obtained from.

Prior to characterizing the plastic particles by SERS, the Raman spectra of both PS and PET were taken by drop casting the stock solutions on a glass slide (Figure A4; Table A2). For PS, key peaks of interest are present as a result of ring breathing vibrations (i.e., v(C–C) near 1002 cm^−1^ and β(C–H) near 1032 cm^−1^) of the benzenes within the polymer backbone [16]. Key peaks of interest for PET are known to be present at 1615–1620 cm^−1^ and 1730 cm^−1^ as a result of the ring breathing and carbonyl stretching, respectively [9,31,32].

After initial characterization, the plastic particle samples were analyzed using SERS. Sample concentrations examined for both the PS sub-microparticles (161 nm) and the PS nanoparticles (33 nm) included 100, 40, 20, and 10 µg/mL. Concentrations of 32 and 15 µg/mL of 62 nm PET particles were also analyzed.

**Figure 2:**
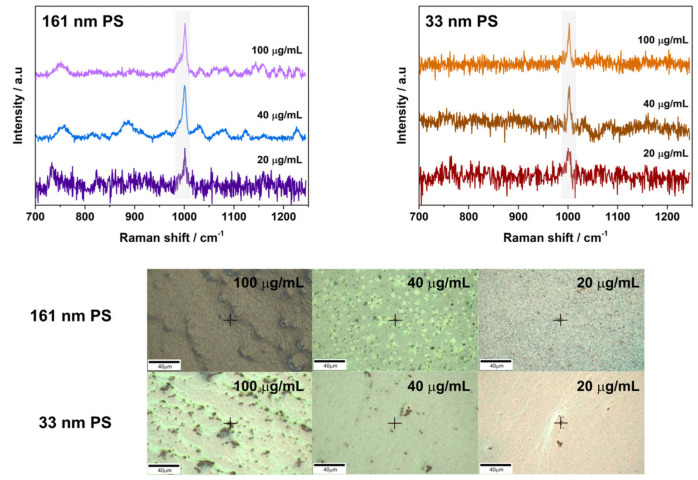
SERS spectra obtained for 161 nm PS particles on 46 nm AuNP substrates (**top**) and 33 nm PS nanoparticles on 46 nm AuNP substrates (**bottom**). Spectrum obtained are shown above images of the region of interest (ROI) the measurement was obtained from. Scale for all is 40 µm. Laser power, scan speeds, and number of averaged accumulations for the samples can be viewed in Table A1.

**Figure A5:**
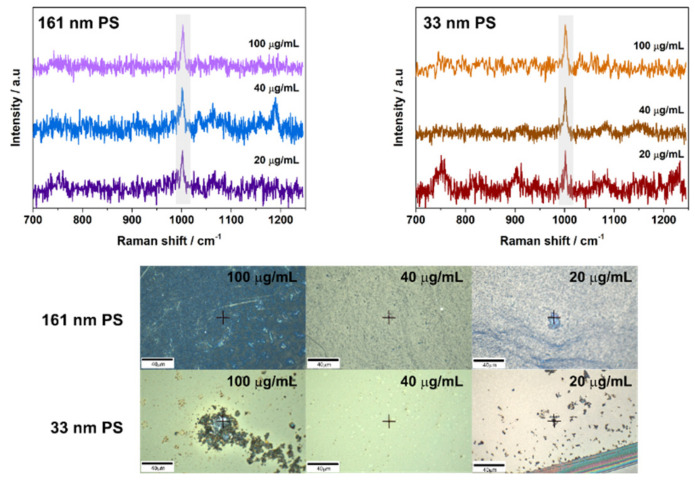
SERS spectra obtained for 161 nm PS particles on 14 nm AuNP substrates as well as the 33 nm PS nanoparticles on 14 nm AuNP substrates. Spectrum obtained are shown above images of the region of interest (ROI) the measurement was obtained from. Scale for all is 40 µm. Laser power, scan speeds, and number of averaged accumulations for the samples can be viewed in Table A1.

**Figure A6:**
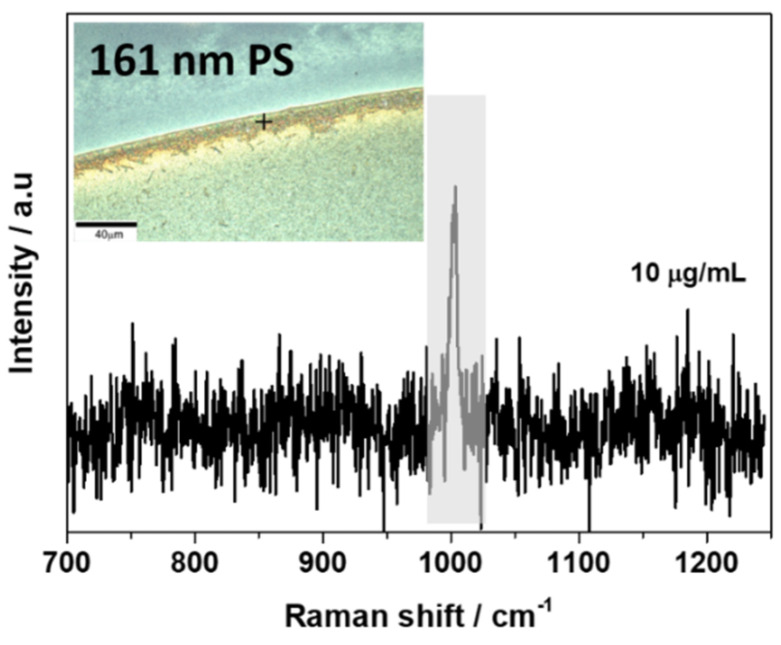
SERS spectra obtained for 10 µg/mL of 161 nm PS particles on 46 nm AuNP substrates. The spectrum is shown with an image of the region of interest (ROI) the measurement was obtained from. Scale is 40 µm. Laser power, scan speeds, and number of averaged accumulations for the samples can be viewed in Table A1.

The 161 nm PS and 33 nm PS samples on 46 nm (Figure 2) and 14 nm (Figure A5) AuNP substrates could be detected down to 20 µg/mL in the center of the cast droplet. Additionally, 161 nm PS could be detected at 10 µg/mL in areas of higher particle concentration (e.g., drop edge, particle aggregates or drying clusters) on the 46 nm AuNP substrates (Figure A6). During these measurements, substrates created using 14 nm AuNPs were shown to have weaker signal than those created using 46 nm AuNPs. Despite the lower enhancements, signal could still be detected on 14 nm AuNP substrates for both sizes of PS particles down to 20 µg/mL (Figure A5). It is important to note that signal for 33 nm PS particles on the 14 nm substrates was found only in regions with high concentration of PS particles (Figure A5). On the 14 nm AuNP substrates, it was not possible to detect 10 µg/mL of plastic particles even at areas of higher particle buildup. For all PS particles, the primary peak of interest used for their detection is present at 1002 cm^−1^ and at the higher sample concentrations (e.g., 100 µg/mL and 40 µg/mL of 161 nm PS), a weak peak at 1032 cm^−1^ can also be observed (Figure 2). Such findings are in good agreement with Raman control data (Figure A4) and findings reported in the literature [16,18].

**Figure 3:**
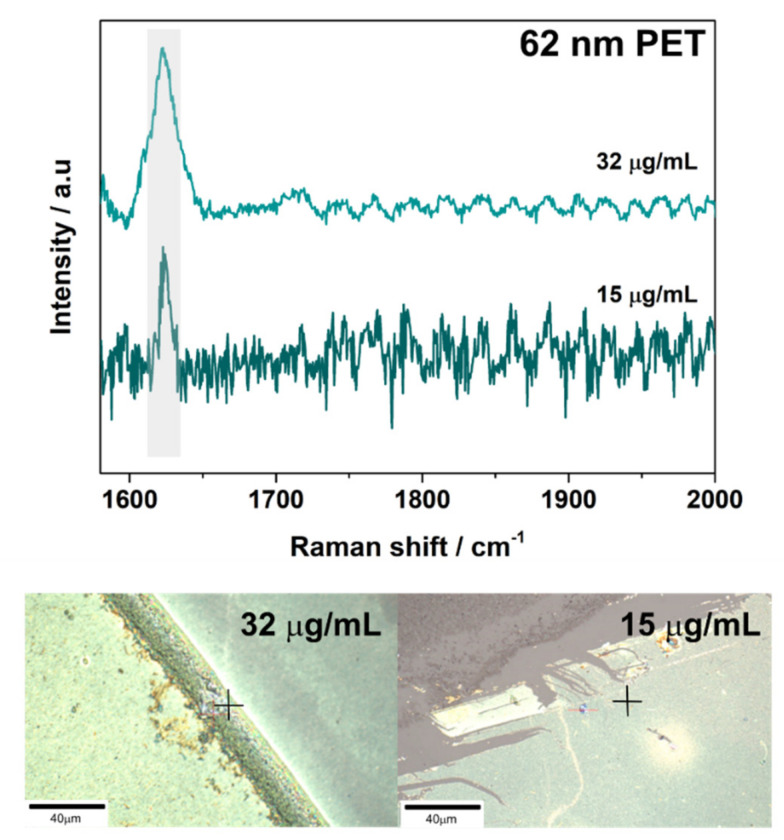
A representative SERS spectrum obtained for 62 nm PET nanoparticles on 46 nm AuNP substrates. The spectrum obtained is shown above images of the region of interest (ROI) the measurement was obtained from. Scale is 40 µm. Laser power, scan speeds, and number of averaged accumulations for the samples can be viewed in Table A1.

**Figure A7:**
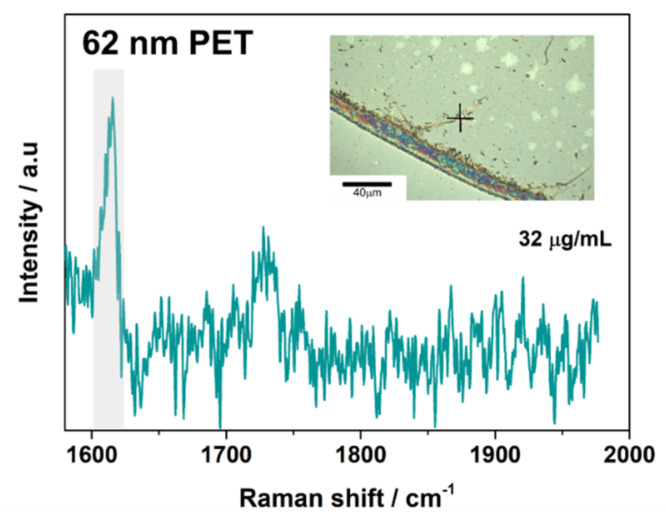
A Representative SERS spectrum obtained for 62 nm PET nanoparticles on 14 nm AuNP substrates. The spectrum is shown with an image of the region of interest (ROI) the measurement was obtained from. Scale is 40 µm. Laser power, scan speeds, and number of averaged accumulations for the samples can be viewed in Table A1.

32 µg/mL of 62 nm PET nanoparticles was detected with both 46 nm and 14 nm AuNP SERS substrates (Figure 3 and Figure A7). Additionally, it was possible to detect PET nanoparticles on 46 nm substrates at a concentration of 15 µg/mL (Figure 3). However, for all PET samples the signal obtained was seen at regions of high particle build-up; indicating the limit of the substrates for PET particle detection was nearly reached during the measurements. This was confirmed when measurements with lower concentrations were attempted on both substrates, but no discernable PET signal could be detected.

**Figure 4:**
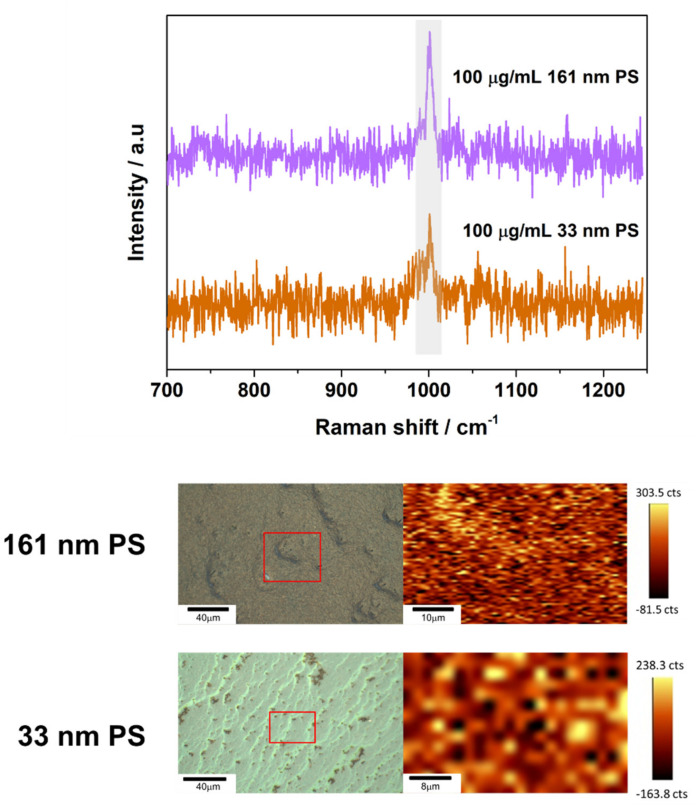
Representative SERS spectra obtained through automated mapping for 100 µg/mL samples of 161 nm PS particles on 46 nm AuNP substrates and samples of 100 µg/mL of 33 nm PS nanoparticles on 46 nm AuNP substrates. Laser power, scan speeds, and number of averaged accumulations for the samples can be viewed in Table A1. Spectrum obtained are shown above images of the intensity maps for the PS peak at 1002 cm^−1^ and the regions of interest (ROIs) the measurement was obtained from. Spectra come from regions of high intensity for the peak at 1002 cm^−1^.

**Figure A8:**
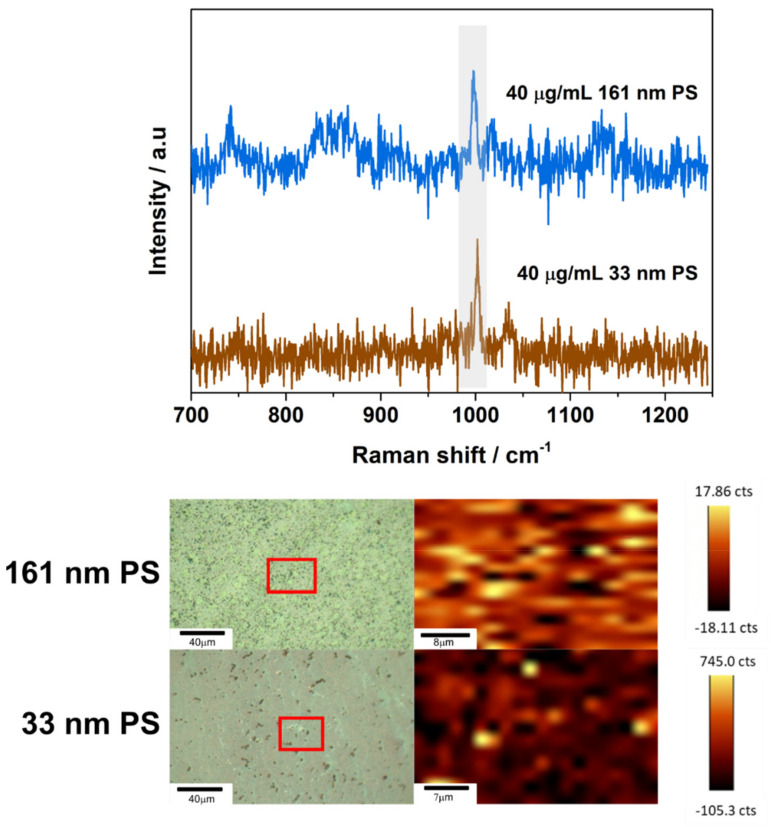
Additional representative SERS spectra obtained through automated mapping for 40 µg/mL samples of 161 nm PS particles on 46 nm AuNP substrates and 40 µg/mL samples of 33 nm PS nanoparticles on 46 nm AuNP substrates. Laser power, scan speeds, and number of averaged accumulations for the samples can be viewed in Table A1. Spectrum obtained are shown above images of the intensity maps for the PS peak at 1002 cm^−1^ and the regions of interest (ROIs) the measurement was obtained from. Spectra come from regions of high intensity for the peak at 1002 cm^−1^.

To further investigate the SERS performance of the fabricated substrates, automated SERS mappings (37 × 27 µm ROI for 40 µg/mL of 33 nm PS; 45 × 30 µm ROI for 100 µg/mL of 33 nm PS; 39 × 28 µm ROI for 40 µg/mL of 161 nm PS; 53 × 46 µm ROI for 100 µg/mL of 161 nm PS) for the 785 nm excitation laser line were performed by recording the SERS intensity at the ring breathing peak of the PS at 1002 cm^−1^. As shown in Figure 4 and Figure A8, the signal intensity obtained in the investigated area is uniform and homogeneous.

**Figure A9:**
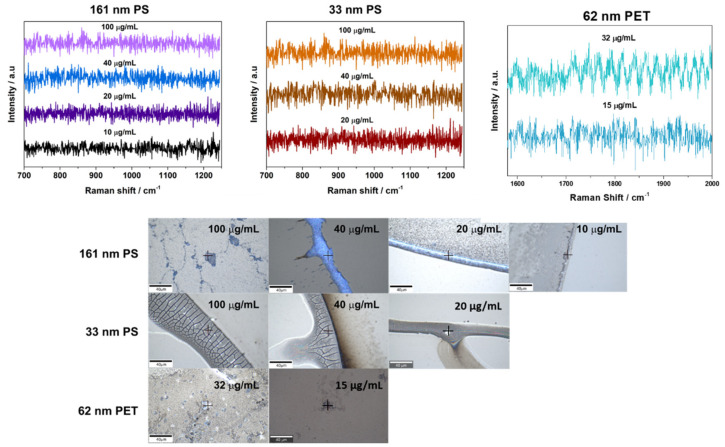
Representative Raman spectra obtained for 161 nm PS particles on plain glass and 33 nm PS nanoparticles on plain glass. Spectra obtained are shown above images of the region of interest (ROI) the measurement was obtained from. The images are labelled with the relevant sample information. Scale for all is 40 µm. Laser power, scan speeds, and number of averaged accumulations for the samples can be viewed in Table A1. Additionally, representative Raman spectrum obtained for PET nanoparticles on plain glass is shown. Scale is 40 µm.

**Figure A10:**
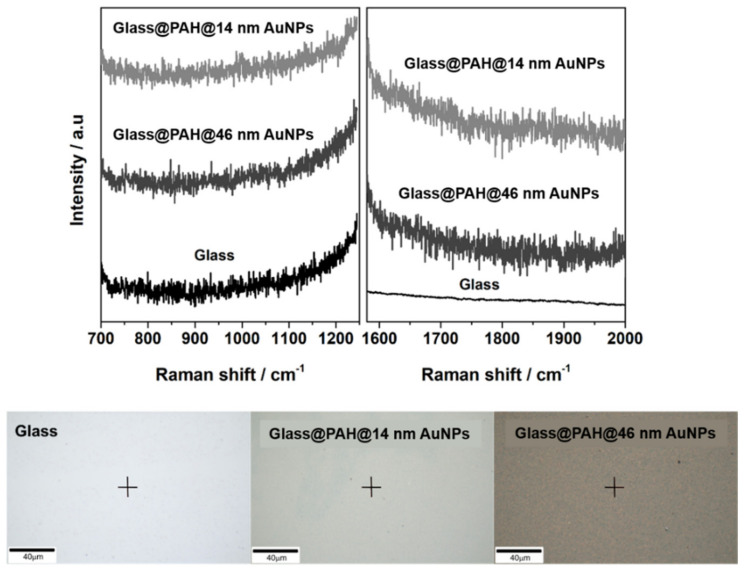
Representative Raman spectra for the substrates and glass slides without the presence of plastic particle samples. The spectra are split into the Raman shift range relevant for PS particles (**left**) and PET particles (**right**). The spectra are shown above images of the ROIs that the spectrum were obtained from, and the images are labelled with the relevant sample title. Laser power, scan speeds, and number of averaged accumulations for the samples can be viewed in Table A1. Scale for all is 40 µm.

It is important to note that no Raman signal was detected for any of the plastic particles at the studied concentrations when they were dried on plain glass; even when areas of high particle concentration (e.g., the sample edge, plastic particle aggregates or drying clusters) were measured. For this comparison, Raman spectra were obtained under conditions comparable to those reported for the SERS measurements (Figure A9). Additionally, the SERS spectra of the plastic particles showed distinct differences from SERS spectra obtained for the substrates alone (Figure A10).

### 3.3. Analytical Enhancement Factor (AEF)

Once SERS spectral data was collected for every sample, the signals obtained could be compared to the control Raman spectra for plastic particles on plain glass (Figure A9). This comparison was done through calculation of the analytical enhancement factor (AEF) achieved for each sample on both types of substrates [32]. The calculation was done using the 1002 cm^−1^ peak (for PS) and the 1617 cm^−1^ peak (for PET) intensity (*I*) and the sample concentration (*C*) both for SERS spectra (*I_SERS_* and *C_SERS_*) and their accompanying Raman control spectra (*I_Raman_* and *C_Raman_*) as shown in Equation (1):

(1) AEF =ISERSCSERSIRamanCRaman

The highest overall AEF was 446 for the 161 nm PS on 46 nm AuNP substrates. For comparison, the highest enhancement obtained for 161 nm PS on 14 nm AuNP substrates was 360. The highest AEF for PET (i.e., 185) was also obtained with the 46 nm AuNP substrates. The 33 nm PS particle samples were the only ones to have the highest AEF (i.e., 127) on the 14 nm AuNP substrates. A complete summary of the AEFs for each sample measured can be found in Table 2.

## 4. Discussion and Conclusions

Through the analysis of plastic particle samples at various concentrations, it was shown that the 46 nm AuNP substrates could detect plastics in samples at lower concentrations than what was possible for the 14 nm AuNP substrates—likely as a result of the increased plasmon bandwidth at increasing AuNP size [33,34]. Thus, the final limits presented for 46 nm AuNP substrates are 10 µg/mL for the 161 nm PS, 20 µg/mL for the 33 nm PS, and 15 µg/mL for the 62 nm PET while the sample concentrations that can be analyzed with the 14 nm AuNP substrates are 20 µg/mL for the 161 nm PS and the 33 nm PS, and 32 µg/mL for the 62 nm PET (Table 2). AEF calculation also revealed that the 46 nm AuNP substrates provided higher signal enhancement when compared to 14 nm AuNP substrates; with maximum AEF for 46 nm AuNP substrates being 446 while the maximum AEF for 14 nm AuNP substrates was 360. This trend is in good agreement with findings previously reported in the literature by groups like Joseph et al. and Zhu et al. [33,35]. Briefly, these two research groups compared the enhancement factors obtained from various sizes of AuNPs; either in dispersion or once they were immobilized on silicon surfaces; and reported an increase in enhancement factor with increasing AuNP size [33,35]. This trend is linked directly to the ability of the AuNPs to scatter light and to the availability of electrons present on the gold surface. Particles below a certain size have surface interactions which become dominated by electronic scattering processes in a manner that diminishes the re-radiated electromagnetic energy and, therefore, the overall SERS signal generated [36]. Additionally, the number of electrons present on the metal surface, and therefore SERS signal, increases with increasing particle size until the size is within a regime comparable to that of the light wavelength used to excite it; at which point only non-radiative plasmon modes are excited [36].

Furthermore, the AEF values reported in this study are comparable to those reported for plastic particles analyzed with other SERS substrates; Xu et al. reported enhancements of up to 2 orders of magnitude for single spherical sub-microplastic particles (i.e., 360 nm) composed of PET, PMMA, or PS on Klarite substrates while Lv et al. reported AgNPs could be used to detect 100 nm PS particles down to 40 µg/mL with AEFs up to 500 [16,18]. In addition to the comparable AEF values, the SERS substrates created in the present study could be used to detect smaller sub-microplastic particles (i.e., 161 nm PS) and nanoparticles (i.e., 33 nm PS or 62 nm PET) at lower concentrations (i.e., down to 10 µg/mL) than what has previously been reported in the literature. For samples with concentrations of 40 µg/mL or higher, the substrates created in this study could be used in automated area scan measurements to generate intensity maps that gave insight into the spatial distribution of the plastic particles.

While it was possible to detect even highly heterogenous nanoplastic particles obtained from milling, the substrates presented herein have not yet been tested for use in the detection of plastics directly within more complex sample matrices (i.e., foods or environmental samples) that would be more relevant to current micro-, sub-micro-, and nanoplastic studies. Thus, future research should consider the analysis of such samples. Additionally, while the concentrations utilized within this study fall within the ranges reported for use in in-vivo laboratory studies conducted with ~70 nm PS nanoparticles (i.e., 155 mg/L–32 mg/L) they are not yet as low as the roughly 1 µg/L–1 ng/L values predicted for environmental samples [6,37]. Thus, further consideration should also be given to the potential use of AuNPs with varying shapes (e.g., rods, stars) that may provide additional signal enhancement [19,38]. However, this study stands as a proof-of-concept for the detection of nanoplastic particles through the use of SERS substrates created using colloidal AuNPs.

## Figures and Tables

**Table 1:** Physicochemical characterization of the particles.

	Core Size ^2^ (nm)	Hydrodynamic Diameter (nm)	Zeta Potential (mV)
46 nm AuNPs	46 ± 5	58 ± 1	−13.3 ± 2.5
14 nm AuNPs	14 ± 1	23 ± 1	−40.2 ± 2.5
161 nm PS particles	161 ± 15	192 ± 4	−45.1 ± 2.3
33 nm PS particles	33 ± 6	40 ± 1	−16.9 ± 1.8
62 nm PET ^1^ particles	62 ± 38	146 ± 1	−28.6 ± 1.0

^1^ Data adapted from Caldwell et al. *Env. Sci.: Nano.* 2021. [22]. ^2^ Core sizes were obtained from electron microscopy measurements of at least 100 representative particles. For AuNPs a TEM was utilized. For plastic particles, a SEM was utilized.

**Table 2:** A summary of analytical enhancement factors (AEFs) calculated for each sample.

	AEF on 46 nm AuNPs	AEF on 14 nm AuNPs
100 µg/mL of 161 nm PS	63.6	23.2
40 µg/mL of 161 nm PS	445.7	50.9
20 µg/mL of 161 nm PS	380.0	360.0
10 µg/mL of 161 nm PS	37.0	----
100 µg/mL of 33 nm PS	97.5	13.6
40 µg/mL of 33 nm PS	53.1	23.0
20 µg/mL of 33 nm PS	56.7	126.7
32 µg/mL of 62 nm PET	185.4	63.1
15 µg/mL of 62 PET	32.8	----

## Data Availability

All raw data used to create the presented figures and tables can be found at http://doi.org/10.5281/zenodo.4722747 (accessed on 27 April 2021).

## Appendix A. Expanded Characterization Details

**Table A1:** A summary of measurement parameters utilized to obtain the final spectra presented for each sample.

Sample	Substrate	Laser Power (mW)	Scan Speed (s)	Number of Accumulations
161 nm PS Raman Control	Glass	40	1	60
100 µg/mL of 161 nm PS	46 nm AuNPs	7	1	300
100 µg/mL of 161 nm PS-Mapping	46 nm AuNPs	7	1	_
100 µg/mL of 161 nm PS	14 nm AuNPs	5	1	40
100 µg/mL of 161 nm PS	Glass	45	1	50
40 µg/mL of 161 nm PS	46 nm AuNPs	7	1	100
40 µg/mL of 161 nm PS-Mapping	46 nm AuNPs	4	1	_
40 µg/mL of 161 nm PS	14 nm AuNPs	4	1	40
40 µg/mL of 161 nm PS	Glass	7	1	50
20 µg/mL of 161 nm PS	46 nm AuNPs	12	1	100
20 µg/mL of 161 nm PS	14 nm AuNPs	12	1	100
20 µg/mL of 161 nm PS	Glass	15	1	100
10 µg/mL of 161 nm PS	46 nm AuNPs	7	1	100
10 µg/mL of 161 nm PS	Glass	45	1	100
100 µg/mL of 33 nm PS	46 nm AuNPs	2	1	25
100 µg/mL of 33 nm PS-Mapping	46 nm AuNPs	2	1	_
100 µg/mL of 33 nm PS	14 nm AuNPs	20	1	100
100 µg/mL of 33 nm PS	Glass	2	1	50
40 µg/mL of 33 nm PS	46 nm AuNPs	2	1	50
40 µg/mL of 33 nm PS-Mapping	46 nm AuNPs	2	1	_
40 µg/mL of 33 nm PS	14 nm AuNPs	7	1	25
40 µg/mL of 33 nm PS	Glass	2	1	50
20 µg/mL of 33 nm PS	46 nm AuNPs	15	1	100
20 µg/mL of 33 nm PS	14 nm AuNPs	10	1	100
20 µg/mL of 33 nm PS	Glass	45	1	100
PET Microparticle Raman Control	Glass	30	1	25
32 µg/mL of 62 nm PET	46 nm AuNPs	10	1	100
32 µg/mL of 62 nm PET	14 nm AuNPs	8	1	50
32 µg/mL of 62 nm PET	Glass	45	1	100
15 µg/mL of 62 PET	46 nm AuNPs	7	1	100
15 µg/mL of 62 PET	Glass	45	1	100
Glass Slide Only Control–Raman Shift 700–1250 cm^−1^	_	7	1	50
Slide@PAH@46nmAu Control–Raman Shift 700–1250 cm^−1^	_	7	1	50
Slide@PAH@14nmAu Control–Raman Shift 700–1250 cm^−1^	_	7	1	50
Glass Slide Only Control–Raman Shift 1580–2000 cm^−1^	_	12	1	100
Slide@PAH@46nmAu Control–Raman Shift 1580–2000 cm^−1^	_	7	1	50
Slide@PAH@14nmAu Control–Raman Shift 1580–2000 cm^−1^	_	7	1	50

**Table A2:** Summary of the vibrational band assignments for PS and PET.

	Raman Shift (cm^−1^)	Assignment
**PS**	~1002	C–C ring breathing mode [16]
	~1032	C–H in-plane deformation, β(C–H) [16]
**PET**	~1615–1620	Aromatic bending vibrations [8,39]
	~1730	Carbonyl stretching mode [39,40]
